# Clinical Validation of the Shock Index, Modified Shock Index, Delta Shock Index, and Shock Index-C for Emergency Department ST-Segment Elevation Myocardial Infarction

**DOI:** 10.3390/jcm11195839

**Published:** 2022-10-01

**Authors:** Charng-Yen Chiang, Chien-Fu Lin, Peng-Huei Liu, Fu-Cheng Chen, I-Min Chiu, Fu-Jen Cheng

**Affiliations:** 1 Department of Emergency Medicine, Kaohsiung Chang Gung Memorial Hospital, College of Medicine, Chang Gung University, Kaohsiung 833, Taiwan; 2 Department of Emergency Medicine, Chang Gung Memorial Hospital, College of Medicine, Chang Gung University, Taoyuan 33302, Taiwan

**Keywords:** ST-segment elevation myocardial infarction, emergency department, shock index, shock index-C, TIMI risk scales, prognosis, in-hospital mortality

## Abstract

Background: ST-segment elevation myocardial infarction (STEMI) is a leading cause of death worldwide. A shock index (SI), modified SI (MSI), delta-SI, and shock index-C (SIC) are known predictors of STEMI. This retrospective cohort study was designed to compare the predictive value of the SI, MSI, delta-SI, and SIC with thrombolysis in myocardial infarction (TIMI) risk scales. Method: Patients > 20 years old with STEMI who underwent percutaneous coronary intervention (PCI) were included. Receiver operating characteristic (ROC) curve analysis with the Youden index was performed to calculate the optimal cutoff values for these predictors. Results: Overall, 1552 adult STEMI cases were analyzed. The thresholds for the emergency department (ED) SI, MSI, SIC, and TIMI risk scales for in-hospital mortality were 0.75, 0.97, 21.00, and 5.5, respectively. Accordingly, ED SIC had better predictive power than the ED SI and ED MSI. The predictive power was relatively higher than TIMI risk scales, but the difference did not achieve statistical significance. After adjusting for confounding factors, the ED SI > 0.75, MSI > 0.97, SIC > 21.0, and TIMI risk scales > 5.5 were statistically and significantly associated with in-hospital mortality of STEMI. Compared with the ED SI and MSI, SIC (>21.0) had better sensitivity (67.2%, 95% CI, 58.6–75.9%), specificity (83.5%, 95% CI, 81.6–85.4%), PPV (24.8%, 95% CI, 20.2–29.6%), and NPV (96.9%, 95% CI, 96.0–97.9%) for in-hospital mortality of STEMI. Conclusions: SIC had better discrimination ability than the SI, MSI, and delta-SI. Compared with the TIMI risk scales, the ACU value of SIC was still higher. Therefore, SIC might be a convenient and rapid tool for predicting the outcome of STEMI.

## 1. Introduction

ST-segment elevation myocardial infarction (STEMI) is one of the leading causes of death worldwide and is mostly induced by an acute reduction in coronary artery blood flow [1]. Despite improvements in percutaneous coronary intervention (PCI) and management of subsequent cardiogenic shock, the in-hospital mortality rate is 2.9–4.7% [2,3]. Many factors are known to be associated with STEMI outcomes, such as sex, age, comorbidities, PCI, and presenting with cardiogenic shock [4,5]. Recently, some studies have attempted to establish risk scales to predict the outcomes of STEMI, such as the thrombolysis in myocardial infarction (TIMI) risk scales and the Global Registry of Acute Coronary Events (GRACE) scales [6,7].

The shock index (SI), calculated from the heart rate (HR) divided by systolic blood pressure (SBP), is a simple tool used to evaluate short-term outcomes of many types of diseases, such as major trauma, critical illness, severe sepsis, and myocardial infarction (MI) [8,9,10,11]. The modified SI (MSI), calculated from HR divided by mean arterial pressure (MAP), was also found to have a predictive value for outcomes of sepsis and coronavirus disease (COVID-19) [12]. On the other hand, Huang et al. demonstrated that the delta-SI, calculated by subtracting the SI at admission from the SI at emergency department (ED) triage, was associated with in-hospital mortality in critically ill patients [9]. Further, many previous studies have shown that a high SI is associated with the risk of in-hospital mortality and major adverse cardiac events (MACEs) in patients with MI [8,13,14]. Furthermore, Shangguan et al. revealed that the MSI has a better predictive value for seven-day mortality and MACEs in STEMI when compared with SI [15]. Recently, Ran et al. established a new score, the shock index-C (SIC; (SI × 100) estimated creatinine clearance rate (CCr)), and found that SIC had a better predictive value than the SI and even better than traditional risk scales, such as TIMI risk scales [16]. However, there is a lack of evidence comparing SIC, the SI, MSI, and delta-SI in predicting the short-term outcomes of STEMI. Moreover, few studies have compared the predictive value of the SI to that of other traditional risk scales for STEMI.

Therefore, the present study had two objectives: (1) to assess the predictive value of the SI, MSI, delta-SI, and SIC in ED patients with STEMI and (2) to compare the predictive value of the SI, MSI, delta-SI, and SIC with TIMI risk scales.

## 2. Materials and Methods

### 2.1. Study Design and Population

This retrospective observational study was conducted between 1 January 2012 and 31 December 2019 in an urban tertiary medical center with an average of 72,000 ED visits per year. The medical records of non-trauma patients older than 20 years who had visited the ED with a principal diagnosis of MI were extracted from the ED administrative database (CD-10: I21.0–I21.3). Two trained emergency physicians reviewed the medical records. The diagnosis of STEMI was made based on the American College of Cardiology/American Heart Association guidelines [17] (see Supplementary Materials) and confirmed by trained cardiologists. Accordingly, patients with STEMI who underwent PCI were included for further analysis. We excluded patients who were transferred to our hospital or who underwent PCI in other centers.

### 2.2. Variables and Outcome Measures

Data on age, sex, triage status, and risk factors for STEMI, including comorbidities such as hypertension, diabetes, and history of MI, were collected from medical records [5]. Biochemical results, PCI findings, and clinical presentations were also extracted. Further, vital signs, including HR, blood pressure, and heart rate, were obtained from the medical records at triage and hospital admission. The SI was calculated by dividing the heart rate (bpm) by the systolic blood pressure (mmHg). The MSI was calculated by dividing the heart rate (bpm) by the mean blood pressure (mmHg). The delta-SI was calculated by subtracting the triage SI from admission SI. In this regard, the delta-MSI was calculated by subtracting the triage MSI from the admission MSI. SIC was defined as (SI × 100)-estimated creatinine clearance (CCr). The estimated creatinine clearance (CCr) was calculated using the Cockcroft–Gault published equations (men: (140–age)/serum creatinine; women: (140–age)/serum creatinine × 0.85) [18]. TIMI risk scales were calculated based on a previously published study [6]. The major outcome was the in-hospital mortality. This study was approved by the institutional review board of Chang Gung Memorial Hospital (number: 202000548B0) and was carried out in accordance with the Code of Ethics of the World Medical Association (Declaration of Helsinki).

### 2.3. Data Analysis

Normally distributed continuous data are presented as mean ± standard deviation (SD), and continuous data with non-normal distribution are presented as median with 25 and 75 percentiles. The independent *t*-test and Mann–Whitney test were used to examine the difference in the distribution of continuous variables. Further, the chi-square test for independence was used to assess differences in categorical variables. A receiver operating characteristic (ROC) curve analysis with the Youden index was used to calculate the optimal cutoff values for the SI, MSI, SIC, and TIMI risk scales. Area under the curve (AUC) values were compared using the DeLong test as previously described [19]. Additionally, the net reclassification improvement (NRI) and integrated discrimination improvement (IDI) were calculated. Further, odds ratios (ORs), 95% confidence intervals (CIs), and *p*-values were calculated using a logistic regression. We assessed the classification performance of these cutoff values using 2 × 2 contingency tables to generate estimates for sensitivity, specificity, positive predictive value (PPV), and negative predictive value (NPV). We calculated 95% confidence intervals (CIs) for each proportion using the method described by Newcombe and Robert [20]. A P significance was set at *p* < 0.05. All statistical analyses were performed using SPSS version 25.0 (IBM Corp., Armonk, NY, USA).

## 3. Results

### 3.1. Characteristics of Study Subjects

During the study period, 1598 patients with STEMI met the STEMI criteria. Fifteen patients were excluded because they were transferred to other hospitals after PCI, nine patients were excluded due to discharge against advice, and twenty-two patients were excluded as they died during PCI. The remaining 1552 patients were included in the further analysis. The demographic characteristics, Killip classification [21] (see Supplementary Materials), and biochemistry results of the 1552 patients are listed in Table 1. There were 116 (7.5%) cases who died during hospitalization. Accordingly, the patients who survived and were discharged from the hospital were mostly men (*p* < 0.001) with younger age (*p* < 0.001) and lower frequency of diabetes (*p* < 0.001), currently smoking (*p* < 0.001), and dyslipidemia (*p* = 0.003). Additionally, the prevalence of previous MI was lower (*p* = 0.023), and patients had a lower Killip class (*p* < 0.001), less mechanical circulatory support (*p* < 0.001), less extracorporeal membrane oxygenation (ECMO) intervention (*p* < 0.001), lower levels of troponin I (*p* < 0.001), and less fatal arrhythmia (*p* < 0.001).

### 3.2. Association between the SI, MSI, Delta-SI, SIC, TIMI Risk Scales and Patients’ Outcome

The calculated SI, MSI, SIC, and TIMI risk scales at ED and hospital admission are listed in Table 2. In this regard, patients who died in the hospital were associated with a higher SI, MSI, and SIC at the ED and hospital admission, as well as higher TIMI risk scales (all *p* values less than 0.001). The differences in positive delta-SI (*p* = 0.333) and delta-MSI (*p* = 0.759) did not reach statistical significance.

**Figure 1:**
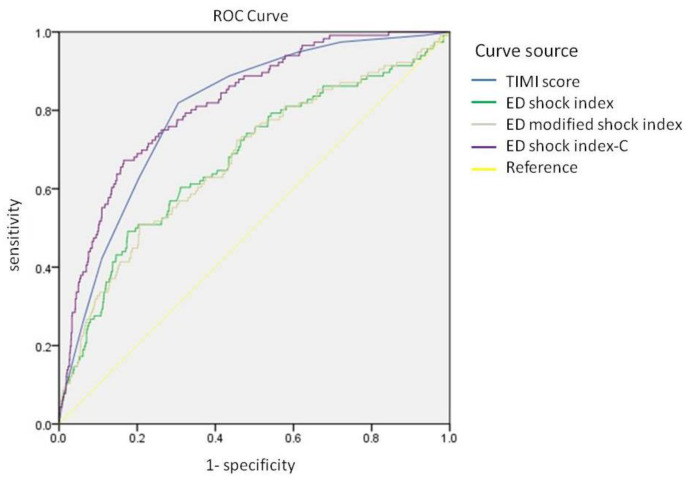
ROC curves of the ED shock index, ED modified shock index, ED shock index-C, and TIMI risk scores.

Table 3 and Figure 1 show the results of the ROC curve analysis and the calculated optimal cutoff values of the SI, MSI, SIC, and TIMI risk scales to predict in-hospital mortality. The thresholds for the ED SI, MSI, SIC, and TIMI risk scales for in-hospital mortality were 0.75 (AUC = 0.676, 0.620–0.731), 0.97 (AUC = 0.674, 0.620–0.729), 21.00 (AUC = 0.818, 0.780–0.858), and 5.5 (AUC = 0.801, 0.764–0.839), respectively. Accordingly, the AUC values of the SI, MSI, and SIC, were higher in ED than that of at admission. ROC curves of the ED SI, MSI, SIC, and TIMI risk scales are shown in Figure 1. The AUC values were compared using the DeLong test, and the results showed that the ED SIC had better predictive power than that of the ED SI (*p* < 0.001; NRI = 93.2%, 95% CI: 76.8−109.7%, *p* < 0.001; IDI = 8.2%, 95% CI: 6.4−10.0%, *p* < 0.001), and ED MSI (*p* < 0.001; NRI = 88.2%, 95% CI: 71.3–105.1%, *p* < 0.001; IDI = 8.1%, 95% CI: 6.3–10.0%, *p* < 0.001). Accordingly, the predictive power was relatively higher than TIMI risk scales (AUC: 0.818 vs. 0.801, *p* = 0.382; NRI = 11.9%, 95% CI: −7.0–30.8%, *p* = 0.218; IDI = 2.9%, 95% CI: 0.1–5.7%, *p* = 0.042), but the difference did not achieve statistical significance. In our cohort, 144 patients had a history of chronic kidney disease (CKD), and 40 patients died during hospitalization. An ROC curve analysis was performed to evaluate the predictive value of the ED SIC, ED SI, and ED MSI. We found that the AUC values (95% CI) of the ED SIC, ED SI, and ED MSI for patients with CKD were 0.618 (0.514–0.721, *p* = 0.029), 0.571 (0.463–0.679, *p* = 0.188), and 0.573 (0.463–0.682, *p* = 0.177), respectively (Supplementary Figure S1).

The AUC curves were compared using the DeLong test, and ED SIC had better predictive power than that of the ED SI (*p* < 0.001) and ED MSI (*p* < 0.001). The predictive power was higher than that of the TIMI risk scale (AUC: 0.818 vs. 0.801); however, the difference was not statistically significant (*p* = 0.382).

### 3.3. Odds Ratio Using Cutoff Values of the SI, MSI, SIC, and TIMI Risk Scales

Table 4 shows the results of the binary logistic regression using cutoff values considering the Youden index. After adjusting for sex, age, diabetes, current smoking status, dyslipidemia, history of myocardial infarction, mechanical circulatory support, ECMO intervention, levels of troponin I, fatal arrhythmia, and high Killip-class, the ED SI > 0.75 (OR = 2.609, 95% CI: 1.649–4.129, *p* < 0.001), MSI > 0.97 (OR = 1.689, 95% CI: 1.057–2.697, *p* = 0.028), SIC > 21.0 (OR = 4.058, 95% CI: 2.515–6.547, *p* < 0.001), and TIMI risk scales > 5.5 (OR = 3.614, 95% CI: 2.016–6.480, *p* < 0.001) showed statistically significant association with in-hospital mortality of patients with STEMI.

### 3.4. Sensitivity, Specificity, Positive Predictive Value, and Negative Predictive for the SI, MSI, SIC, and TIMI Risk Scales

Table 5 displays a two-by-two contingency table from which we generated the cutoff values of the ED SI, MSI, SIC, and TIMI risk scales. Compared with the ED SI and MSI, SIC (>21.0) had better sensitivity (67.2%, 95% CI, 58.6–75.9%), specificity (83.5%, 95% CI, 81.6–85.4%), PPV (24.8%, 95% CI, 20.2–29.6%), and NPV (96.9%, 95% CI, 96.0–97.9%) for in-hospital mortality of patients with STEMI. Accordingly, when the cutoff value of the TIMI risk scale was set to 5.5, we documented a sensitivity of 81.9% (95% CI, 74.8–89.0%), specificity of 69.6.0% (95% CI, 67.2–72.0%), PPV of 17.9% (95% CI, 14.6–21.1%), and NPV of 97.9% NPV (95% CI, 97.1–98.8%).

## 4. Discussion

In the present study, compared with the SI, MSI, and delta-SI, ED SIC had a better predictive value for in-hospital mortality in STEMI patients. The AUC value of SIC for in-hospital mortality was better than that of the TIMI risk scales, but the difference was not statistically significant. In our cohort, the optimal cutoff value for ED SIC was 21, with 83.5% (81.6–85.4%) specificity and 96.9% (96.0–97.9%) negative predictive value. Furthermore, compared to the TIMI risk scales, SIC was more convenient to obtain and calculate and had better specificity for predicting in-hospital mortality for patients with STEMI. Our results suggest that ED SIC is a convenient and rapid tool for predicting in-hospital mortality in STEMI patients undergoing PCI.

Previous studies have shown that the SI is associated with MI outcomes. Abe et al. analyzed 610 STEMI patients who underwent PCI and found that an elevated SI (>0.66) was associated with a higher risk of five-year MACEs [22]. Kobayashi et al. collected data from 481 patients with non-ST-segment elevation MI and revealed that a higher SI (>0.7) was related to a higher risk of in-hospital mortality and cardiogenic shock [14]. Another study analyzed data from 7412 STEMI patients receiving PCI and demonstrated that elevated admission SI (≥0.7) was a predictor of 30-day and 1-year mortality rates [8]. Further, a review article collected eight studies on acute MI and concluded that a high SI (>0.7) was associated with the risk of in-hospital mortality and long-term adverse outcomes [23]. In this regard, the MSI has also been used to predict the outcomes of MI. Abreu et al. analyzed data from patients with STEMI and found that a higher MSI (≥0.93) was a predictor of in-hospital fatal arrhythmia and six-month mortality [24]. Accordingly, another study collected data from both STEMI and non-STEMI patients and compared the predictive value of the SI and MSI for long-term (median observational time of 6.5 years) outcomes. They revealed that an elevated SI and MSI were indicators of poor outcome, and the MSI seemed to have a better predictive value than the SI for non-STEMI [25]. In this regard, the SI and MSI appear to be predictors of short- and long-term outcomes for acute MI. In the present study, we also found that elevated SI and MSI levels were associated with an increased risk of in-hospital mortality.

A delta-SI was initially applied to trauma patients as a predictor of in-hospital mortality, and a positive delta-SI (ED SI minus field SI) was an independent predictor of mortality [26,27]. Asmar et al. used a delta-SI to predict prognosis for pediatric traumatic patients, and they revealed that a delta-SI was associated with the risk of 24-h mortality and in-hospital mortality [28]. Another study compared the predictive value of the delta-SI and SI on postpartum hemorrhage and showed a better power for the delta-SI in predicting the need for blood transfusion and surgical intervention [29]. Huang et al. recorded vital signs from the ED and intensive care unit admission to calculate the delta-SI and evaluate the association between a delta-SI and in-hospital mortality in critically ill patients. They found that a positive delta-SI was associated with the risk of mortality within 48 h and in-hospital mortality [9]. However, they did not compare the predictive values between the SI and delta-SI. In our cohort, we did not find a statistically significant difference in the delta-SI between the survival and mortality groups for STEMI patients. Our results suggest that the SI, MSI, and SIC are better predictors of STEMI outcome when compared with a delta-SI.

In 2021, Ran et al. developed SIC to predict the outcome of STEMI because renal function was one of the major factors influencing the outcome of STEMI [16]. They found that the predictive value of SIC was better than that of the SI and TIMI risk scales. This study is the first to compare SIC with the SI, MSI, and delta-SI. According to our results, SIC had better predictive power than the SI, MSI, and delta-SI for STEMI. Additionally, our results revealed a better AUC value for SIC than that of the TIMI risk scales. Furthermore, compared with TIMI risk scales, SIC is a more convenient tool, and the NPV may be acceptable. Therefore, SIC may be a rapid tool for predicting the outcome of STEMI.

While we made a detailed comparison of predictive formulas, the SI has been the subject of most studies in conditions including trauma, myocardial injury, and sepsis. It is valuable in predicting hemorrhagic shock requiring the activation of a massive transfusion protocol (MTP) compared to traditional measures such as hypotension or tachycardia because of its ability to identify patients in the compensatory phase of shock [30]. In addition, Bilkova et al. found that the SI is a strong independent predictor of in-hospital mortality in patients with STEMI [31]. Similarly, Hwang et al. found that in the group of STEMI patients undergoing primary PCI, an SI > 0.7, compared with an SI ≤ 0.7, is associated with a larger myocardial infarct size and a higher hemorrhagic infarction rate [32]. Additionally, some studies have indicated that the initial elevated SI can also be a moderately accurate predictor of mortality in adult patients with suspected sepsis [33,34].

While a modified MSI was found to be superior to the SI in predicting mortality in ED studies, Liu et al. indicated that there is an increased probability of intensive care unit (ICU) admission and death when the MSI is >1.3 [35]. Similarly, Althunayyan et al. also reported that an MSI  ≥  1.3 could be a good predictor for sepsis-related outcomes, including hyperlactatemia, ICU admission, and 28 days mortality [36]. Recently, it has been noted that other indices, such as delta-SI, could predict mortality in patients with trauma or postpartum hemorrhage [26,27]. Further research also indicated that delta-SI appeared to be an effective and efficient index of great relevance for rapid deterioration after ED admission [9].

Considering the scales for patients with acute coronary syndrome, several risk scores have been developed for predicting the outcomes in this group of patients such as GRACE and TIMI scores [37]. A positive correlation between these two risk scores and angiographic severity of non-ST-elevation acute coronary syndrome has been noted [38,39]. Regarding the application in the group of STEMI patients, a previous study has shown that the TIMI risk score reliably identifies patients in both high-risk and low-risk ranges [38]. The GRACE score can also improve patient selection for clinical and interventional procedures following STEMI episodes [40]. Accordingly, the GRACE risk score can be helpful in guiding cardiologists in selecting the appropriate time for coronary intervention in STEMI patients who had received fibrinolytic treatment [41]. Although these two scores show similar discriminatory capacities for hospital death, some studies have pointed out that the TIMI score has better calibration than GRACE [42].

In conclusion, SIC had better discrimination ability than the SI, MSI, and delta-SI. Compared with the TIMI risk scales, the ACU value of SIC was still higher. Therefore, SIC might be a convenient and rapid tool for predicting the outcome of STEMI. Moreover, one important and obvious advantage of SIC is that it includes three simple data (heart rate, systolic blood pressure, and CCr) that are easily gathered and calculated. Therefore, SIC can be used to rapidly and precisely assess and stratify the risk of multiple events, without additional effort.

## 5. Conclusions

In our cohort, the SI, MSI, and SIC were independently associated with in-hospital mortality in STEMI patients who underwent PCI. We found that SIC had better discrimination ability than the SI, MSI, and delta-SI. Accordingly, compared with the TIMI risk scales, the AUC value of SIC was higher. Moreover, one important and obvious advantage of SIC is that it includes three simple data (heart rate, systolic blood pressure, and CCr) that are easily gathered and calculated. As a result, SIC may be a rapid tool for assessing and stratifying STEMI outcomes.

## 6. Limitation

This study had some limitations. First, this was a retrospective observational study limited to one city with a single hospital. Our findings may not be generalizable to other locations or hospitals with different medical systems. Second, prehospital interventions and drugs prescribed before the ED visit were not recorded in the database. Third, our study did not include long-term outcomes, such as long-term survival or long-term MACEs. Fourth, post-PCI care, such as intra-aortic balloon pump or extracorporeal membrane oxygenation interventions, were not included in the analysis.

## Figures and Tables

**Table 1:** Demographic characteristics of 1552 STEMI patients receiving PCI.

	Survival to Hospital Discharge	Mortality	*p*
Characteristics of ST-elevation myocardial infarction	*n* = 1436	*n* = 116	
Age	60.3 ± 12.6	69.3 ± 12.8	<0.001
Male sex	1217	80	<0.001
Body mass index	25.6 ± 3.9	25.3 ± 4.6	0.466
Hypertension	938	76	0.966
Diabetes	550	62	0.001
Current smoker	812	43	<0.001
Dyslipidemia	1103	75	0.003
History of myocardial infarction	121	17	0.023
History of PCI	122	16	0.054
Killip II to IV	506	103	<0.001
Anterior wall STEMI	752	60	0.894
Mechanical circulatory support	194	77	<0.001
ECMO intervention	15	34	<0.001
Troponin I	3.8 ± 12.6	13.7 ± 24.7	<0.001
Fatal arrhythmia	104	37	<0.001

STEMI, ST-elevation myocardial infarction; PCI, percutaneous coronary intervention; ECMO, extracorporeal membrane oxygenation.

**Table 2:** Calculated SI, MSI, SIC, and TIMI risk scales at ED and hospital admission of STEMI patients receiving PCI.

	Survival to Hospital Discharge (*n* = 1436)	Mortality (*n* = 116)	*p*
ED shock index	0.57 (0.46, 0.71)	0.74 (0.57, 0.93)	<0.001
ED modified shock index	0.76 (0.63, 0.93)	0.97 (0.76, 1.25)	<0.001
ED mean arterial pressure	102 (87, 119)	91.5 (73.8, 109.3)	<0.001
ED shock index-C	−16.2 (−39.3, 8.5)	35.2 (5.5, 59.0)	<0.001
TIMI risk scores	4 (2, 6)	7 (6, 9)	<0.001
Admission shock index	0.65 ± 0.21	0.81 ± 0.37	<0.001
Admission modified shock index	0.85 (0.72, 1.01)	1.04 (0.83, 1.28)	<0.001
Admission mean arterial pressure	98 (86, 110)	88 (70, 107)	<0.001
Admission shock index-C	−10.3 (−33.9, 13.3)	32.7 (5.4, 55.3)	<0.001
Positive delta shock index	578	39	0.333
Positive delta modified shock index	573	42	0.759

STEMI, ST-elevation myocardial infarction; SI, shock index; MSI, modified shock index; SIC, shock index-C; TIMI, thrombolysis in myocardial infarction; ED, emergency department; PCI, percutaneous coronary intervention.

**Table 3:** Receiver operating characteristic (ROC) curve analysis of the optimal thresholds of the SI, MSI, SIC, and TIMI risk scores for predicting in-hospital mortality.

Overall					
	Survival to Hospital Discharge				
	Threshold	AUC	Lower	Upper	*p*
ED Shock index	0.75	0.676	0.620	0.731	<0.001
ED Modified shock index	0.97	0.674	0.620	0.729	<0.001
ED shock index-C	21.00	0.818	0.780	0.856	<0.001
Admission shock index	0.75	0.662	0.605	0.719	<0.001
Admission modified shock index	1.03	0.670	0.611	0.730	<0.001
Admission shock index-C	28.00	0.792	0.748	0.836	<0.001
TIMI risk scores	5.50	0.801	0.764	0.839	<0.001

AUC, area under the curve; ED, emergency department; TIMI, thrombolysis in myocardial infarction.

**Table 4:** Odds ratio (OR) and 95% confidence interval (95% CI) for in-hospital mortality in STEMI patients after adjusting for sex, age, diabetes, current smoking status, dyslipidemia, history of myocardial infarction, mechanical circulatory support, ECMO intervention, level of troponin I, fatal arrhythmia, and high Killip score.

Adjusted Odds Ratios for In-Hospital Mortality				
	OR	95% CI		*p*
ED shock index > 0.75	2.609	1.649	4.129	<0.001
ED modified shock index > 0.97	1.689	1.057	2.697	0.028
ED shock index-C > 21.0	4.058	2.515	6.547	<0.001
TIMI score > 5.5	3.614	2.016	6.480	<0.001
Admission shock index > 0.75	2.759	1.727	4.407	<0.001
Admission modified shock index > 1.03	2.234	1.455	30712	<0.001
Admission shock index-C > 28	3.099	1.908	5.034	<0.001
Positive delta shock index	0.757	0.481	1.191	0.229

TIMI, thrombolysis in myocardial infarction; ED, emergency department; OR, odds ratio; CI, confidence interval; ECMO, extracorporeal membrane oxygenation.

**Table 5:** Sensitivity, specificity, positive predictive value, and negative predictive value of the SI, MSI, SIC and TIMI risk scores for in-hospital mortality of STEMI patients; ED, emergency department; TIMI, thrombolysis in myocardial infarction; CI, confidence interval.

Sensitivity, Specificity and Negative Predictive Value of STEMI in Hospital Mortality											
Assessment Using ED Shock Index-C > 21			Assessment Using ED Shock Index > 0.75			Assessment Using ED Modified Shock Index > 1			Assessment Using TIMI Score > 5.5		
Assessment Using Shock Index-C > 21	Survival to Hospital Discharge	Mortality	Assessment Using ED Shock Index > 0.75	Survival to Hospital Discharge	Mortality	Assessment Using ED Modified Shock Index > 1	Survival to Hospital Discharge	Mortality	Assessment Using TIMI Score > 5.5	Survival to Hospital Discharge	Mortality
No. of positive results	237	78	No. of positive results	264	57	No. of positive results	257	48	No. of positive results	437	95
No. of negative results	1198	38	No. of negative results	1171	59	No. of negative results	1178	68	No. of negative results	999	21
Sensitivity, % (95% CI)	67.2 (58.6–75.9)		Sensitivity, % (95% CI)	49.1 (39.9–58.4)		Sensitivity, % (95% CI)	41.4 (32.3–50.5)		Sensitivity, % (95% CI)	81.9 (74.8–89.0)	
Specificity, % (95% CI)	83.5 (81.6–85.4)		Specificity, % (95% CI)	81.6 (79.6–83.6)		Specificity, % (95% CI)	82.1 (84.1–80.1)		Specificity, % (95% CI)	69.6 (67.2–72.0)	
Positive Predictive Value, %	24.8 (20.2–29.6)		Positive Predictive Value, %	17.8 (13.6–22.0)		Positive Predictive Value, %	15.7 (11.6–19.8)		Positive Predictive Value, %	17.9 (14.6–21.1)	
Negative predictive value, %	96.9 (96.0–97.9)		Negative predictive value, %	95.2 (94.0–96.4)		Negative predictive value, %	94.5 (93.3–95.8)		Negative predictive value, %	97.9 (97.1–98.8)	

## Data Availability

Data were obtained from Chang Gung Research Database and are available by corresponding with the author and obtaining permission.

## Supplementary Materials

The following supporting information can be downloaded at: https://www.mdpi.com/article/10.3390/jcm11195839/s1, Figure S1: ROC curves of ED shock index, ED modified shock index, and ED shock index-C for STEMI patients with history of chronic kidney disease. References [17,21] are cited in the supplementary materials.
