# Supplementary material for: Clinical Validation of the Shock Index, Modified Shock Index, Delta Shock Index, and Shock Index-C for Emergency Department ST-Segment Elevation Myocardial Infarction
Source: J Clin Med. 2022 Oct 1;11(19):5839. doi: 10.3390/jcm11195839 (PMC9573755; doi:10.3390/jcm11195839)
Supplement: Supplementary file 1 [file jcm-11-05839-s001.zip › jcm-1908103-supplementary.pdf]

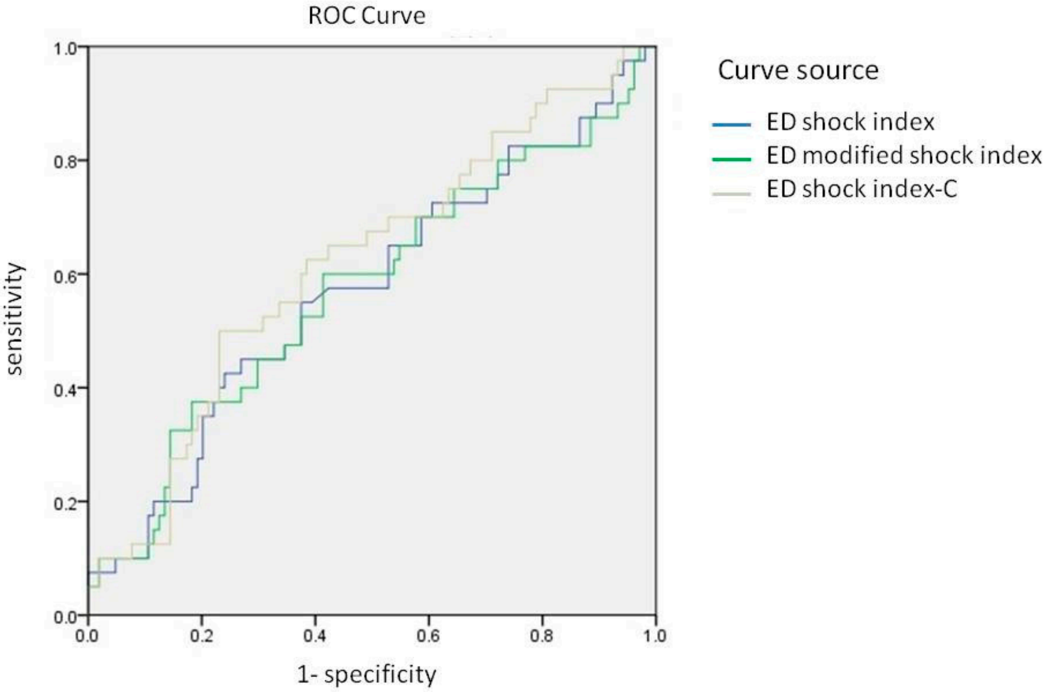

Figure S1. ROC curves of ED shock index, ED modified shock index, and ED shock index-C for STEMI patients with history of chronic kidney disease.

The area under the curve (AUC) value (95% CI) of ED SIC, ED SI, and ED MSI for those with chronic kidney disease were 0.618 (0.514–0.721, $p = 0.029$), 0.571 (0.463–0.679, $p = 0.188$), and 0.573 (0.463–0.682, $p = 0.177$).

Supplementary Materials

1. Killip classification [1]
   Class I, No evidence of heart failure
   Class II, Findings consistent with mild to moderate heart failure (eg, S3 gallop, lung rales less than one-half way up the posterior lung fields, or jugular venous distension)
   Class III, Overt pulmonary edema
   Class IV, Cardiogenic shock
2. Definition and diagnosis for ST-Segment elevation myocardial infarction (STEMI) [2]

STEMI is a clinical syndrome defined by characteristic symptoms of myocardial ischemia in association with persistent electrocardiographic (ECG) ST elevation and subsequent release of biomarkers of myocardial necrosis. Diagnostic ST elevation in the absence of left ventricular (LV) hypertrophy or left bundle-branch block (LBBB) is defined as new ST elevation at the J point in at least 2 contiguous leads of ≥2 mm (0.2 mV) in men or ≥1.5 mm (0.15 mV) in women in leads V2–V3 and/or of ≥1 mm (0.1 mV) in other contiguous chest leads or the limb leads. New or presumably new LBBB has been considered a STEMI equivalent. In addition, ST depression in ≥2 precordial leads (V1–V4) may indicate transmural posterior injury; multi-lead ST depression with coexistent ST elevation in lead aVR has been described in patients with left main or proximal left anterior descending artery occlusion.

References

1. Killip, T., 3rd; Kimball, J.T. Treatment of myocardial infarction in a coronary care unit: A two year experience with 250 patients. *Am. J. Cardiol.* **1967**, *20*, 457–464.
2. O'Gara, P.T.; Kushner, F.G.; Ascheim, D.D.; Casey, D.E., Jr.; Chung, M.K.; de Lemos, J.A.; Ettinger, S.M.; Fang, J.C.; Fesmire, F.M.; Franklin, B.A.; et al. 2013 ACCF/AHA guideline for the management of ST-elevation myocardial infarction: A report of the American College of Cardiology Foundation/American Heart Association Task Force on Practice Guidelines. *Circulation* **2013**, *127*, e362–425.
